# Editorial: Women in plant symbiotic interactions: 2022

**DOI:** 10.3389/fpls.2024.1357641

**Published:** 2024-01-16

**Authors:** Alessandra Salvioli di Fossalunga, Federica Spina

**Affiliations:** Department of Life Sciences and Systems Biology, University of Turin, Turin, Italy

**Keywords:** plant symbiosis, mycorrhiza, endophytes, plant-microbe interactions, fungi

The role of women in science has been for a long time neglected, and their involvement in important discoveries omitted. According to the UNESCO Institute for Statistics, in 2019 less than 30% of the world’s researchers were women. Nevertheless, the number of women engaged in research is continuously increasing, and their contribution in past research is acknowledged, such as their role in revealing the structure of DNA.

To date, the 41% and 43.1% of all scientists are women in Europe and United States, respectively.

The recent story of the symbiosis study has a feminine hallmark. In 1967 Lynn Margulis, with her unconventional theories, challenged the uniqueness of the Darwinian view: she proposed that cooperation, along with competition, is one of the major driving forces in evolution.

The present Research Topic shows different aspects that are considered hot issues in the plant symbiotic field.

Mutualistic relationships in the rhizosphere can be harnessed to improve crops performance in an agronomic perspective: based on this, the use of microbial biostimulants in agriculture is rising fast in recent years. Among the microorganisms employed, arbuscular mycorrhizal fungi (AMF) have pride of place. Ganugi et al. investigated the effect of seven different AMF-based commercial products on an Italian grape cultivar.

High temperature and aridity strongly impact grapevine cultivation, with specific effects on plant photosynthetic efficiency (Arias et al., 2022). The Authors evaluated the effect of the AMF-based treatments analyzing different plant parameters such as the leaf metabolite content, the fruit phenolic profile and the plant photosynthetic performance. The trial has been performed in a very hot and arid season (growing season 2022), and interestingly the AMF-based products were shown to be particularly effective in improving the photosynthetic process under conditions that normally lead to a significant decline of PSII photochemical efficiency. Concerning the leaf metabolic content, glycolipids and phospholipids turned up to be increased upon AMF-based treatments; secondary metabolites such as alkaloids and terpenoids showed opposite trends depending on the treatment considered. The discrepancy in the effect exerted by different treatments on grape quality traits highlights the importance of the choice of suitable products in terms of their composition, timing and dosage of application when a biostimulant-based approach is adopted on cultures.

AMF are considered to some extent elusive microbes to be studied, as they are obligate biotrophs of plants. The mycorrhiza takes time to be established, and can be disturbed by several external factors such as excessive N-P-K fertilization (Kuila and Ghosh, 2022). Soil chemical composition is indeed one of the main drivers of the fungal community structure, being particularly true in trace-element (TE) contaminated soils. Both fungal richness and diversity is affected, but plant development as well (Ciadamidaro et al.). Fungi are not silent spectators but may instead play a central role in plant survival rate. Innovative afforestation techniques take advantage of these powerful fungal-plant interactions and ultimately help to recover contaminated land and restore their ecological services. Inoculation of mycorrhizal consortia including both AMF and ectomycorrhizal fungi (ECM) had positive effects on poplar, otherwise strongly affected by stressful conditions posed by TE contamination (Ciadamidaro et al.). By increasing the surface area available for nutrient and water uptake, plants better tolerated the unfavorable conditions, resulting in higher biomass development.

Water scarcity represents an actual threat for agriculture. Indeed agriculture production depends on water availability but it is also responsible for 24% of water abstraction in Europe only (ECA, 2021). Many plant-beneficial fungi, such as *Trichoderma* spp., are commonly used as control agents against biotic stressors, but could also stimulate abiotic tolerance in crops. *Trichoderma* isolates did indeed mitigate water deficit stress on sensitive cultivars, such as tomatoes (Rawal et al.). Authors elucidated the involved metabolic pathways: fungi did enhance crop tolerance to drought conditions by improving the photosynthetic rate and stomatal conductance, as well as increasing the chlorophyll content. However, as in many other aspects of life, the choice of the correct partner should not be underestimated. *Trichoderma* is not an exception to this axiom. Among the strains tested, only a few isolates established a dual-relation with tomato plants capable of providing strength against water deficit stress. Interestingly, the most performing strains were isolated in the driest region in Nepal, suggesting that this extreme ecological niche may have stimulated peculiar adaptation skills.

Pop-Moldovan et al. applied a new mycorrhiza assessment method on maize roots from a field set up at different plant phenological stages. The main aim of the research was to elucidate the effect of a biostimulant product containing organic compounds on root mycorrhization as supported by native AMF communities in the soil, from the vegetative to the ripening stage of maize plants. The Authors found that the intensity of colonization was reduced in treated versus untreated plants until the end of the vegetation period. The untreated plants had more arbuscules in newly colonized root areas, and for the whole plant vegetative stage. Thanks to the use of their mycorrhiza assessment method and of a robust statistical analysis, the Authors concluded that the reduction of mycorrhization in the biostimulant-treated plants is rather due to a lower colonization of new root areas, that are gradually accessible to the fungus during the whole vegetative period.

Biological nitrogen fixation associated with maize has a great potential to improve plant nutrition, and involves a complex and poorly known microbial community populating the mucilage produced by aerial roots (Pankievicz et al.). The age of the aerial roots and their diameters or the abundance of border cells all affected the nitrogen uptake, but research is far from determining how these factors actually influence the mucilage environment. Authors suggested that additional efforts based on synthetic community and systems biology are needed to enhance the knowledge about the molecular interaction between plants and fungi. Indeed, molecular mycorrhiza research is nowadays a very active field of research, and new analytical techniques are helping clarify the gray areas which remain on this matter.

The establishment and functioning of plant-fungal mutualistic endosymbiosis entails a dense and fine-tuned molecular dialogue which is to date not completely elucidated. During the establishment of the arbuscular mycorrhizal symbiosis, the newly formed arbuscule in the root cell is surrounded by the peri-arbuscular membrane (PAM), of plant origin. The formation of PAM implies the supply of building material for newly forming membrane which is delivered via membrane trafficking. The secretory vesicle delivery is mediated by a group of proteins called SNARE (soluble N-ethylmaleimide-sensitive factor attachment protein receptor). Liu et al. employed CRISPR/Cas9 technique to generate rice mutants for three *Oryza sativa* SNARE genes, of which one was predicted to be involved in the symbiosis (OsSYP132) and two (OsSYP131a and OsSYP131b) constitutively expressed (i.e. non-symbiotic). They observed that homozygous OsSYP131b and OsSYP132 mutant lines displayed apparently normal asymbiotic growth and normal mycorrhization patterns. On the contrary, the OsSYP131a-1 homozygous mutant produced infertile seeds, indicating a possible role of OsSYP13a in rice grain fertility. Interestingly, the OsSYP131b-OsSYP132 double mutant, although showing a normal asymbiotic phenotype, displayed a marked reduction in the fungal colonization rate and arbuscle abundance, probably indicating a less efficient though still working membrane trafficking pathway in rice roots.

Giovannetti et al. reviewed the past, present and future of the soil fertility concept, highlighting how it changed over the years to currently include the biotic component. The Authors urge researchers to develop new and comprehensive indicators to also take into account the multifaceted effects that soil microbiota, including symbiotic microbes, can have on soil to eventually sustain plant (crops) performance. Finally, they propose that traits related to plant health can also be used as proxies to infer different characteristics that feature soil fertility. This is nowadays made possible by the development of new technologies allowing an accurate monitoring of plant traits even under field conditions. Finding universally recognized methods to determine “how good is a soil” based on plant performance is in fact of paramount importance towards a precision agriculture approach that will entail a reduction in phytochemical use, limited to the bare necessities, and a more conscious harnessing of the rhizosphere microbiota.

## Author contributions

ASDF: Writing – original draft, Writing – review & editing. FS: Writing – original draft, Writing – review & editing.
